# Using EEG and MEG to characterize extreme delta brush in a patient with anti-NMDA receptor encephalitis

**DOI:** 10.1186/s12883-021-02157-0

**Published:** 2021-03-22

**Authors:** Ailiang Miao, Yongwei Shi, Jing Xiang, Xiaoshan Wang, Jianqing Ge, Qiqi Chen, Yuanwen Yu, Chuanyong Yu, Di Wu

**Affiliations:** 1 Department of Neurology, The Affiliated Brain Hospital of Nanjing Medical University, Nanjing Medical University, Guang Zhou Road 264, Jiangsu 210029 Nanjing, China; 2 Department of Video-Electroencephalogram, The Affiliated Brain Hospital of Nanjing Medical University, Nanjing Medical University, Jiangsu Nanjing, China; 3 Department of Neurology, Taizhou Fourth People’s Hospital, Jiangsu Taizhou, China; 4 MEG Center, Division of Neurology, Cincinnati Children’s Hospital Medical Center, OH 45220 Cincinnati, USA; 5 MEG Center, Nanjing Brain Hospital, Jiangsu 210029 Nanjing, China

**Keywords:** anti-N-methyl-d-aspartate receptor encephalitis, Electroencephalography, Magnetoencephalography, Extreme delta brush

## Abstract

**Background:**

Extreme delta brush (EDB) is considered a potential marker for anti-N-methyl-d-aspartate receptor (anti-NMDAR) encephalitis. The brain regions involved in EDB are unclear.

**Case presentation:**

A 16-year-old woman with anti-NMDAR encephalitis who was experiencing psychosis was admitted. Electroencephalography (EEG) and magnetoencephalography (MEG) were used to analyze EDB in the patient. EDB on EEG could be disturbed by opening and closing the eyes, by occipital alpha rhythms and by sleep-wake cycles. The MEG results showed beta activity originating from bilateral superior parietal lobes. However, the delta wave originated from bilateral superior temporal gyri, the right middle temporal gyrus, the right inferior frontal gyrus, and the left inferior parietal lobe.

**Conclusions:**

Delta wave and beta activity might originate from different brain regions. Beta activity might be transmitted forward to the frontotemporal lobe and superimposed with delta activity to form EDB on EEG.

## Background

Anti-N-methyl-d-aspartate receptor (anti-NMDAR) encephalitis is an autoimmune disease associated with serum and/or cerebral spinal fluid (CSF) antibodies against functional NMDAR [1, 2]. Patients develop acute or subacute psychiatric symptoms, memory loss, movement disorders, seizures, speech dysfunction and disturbance of consciousness [3].

Electroencephalography (EEG) can be useful for diagnosing anti-NMDAR encephalitis [4–9]. Extreme delta brush (EDB) is a characteristic EEG pattern of anti-NMDAR encephalitis [4]. EDB is consists of rhythmic delta activity at 1–3 Hz with superimposed bursts of rhythmic beta frequency activity “riding” on each delta wave. The beta/delta power ratio and rhythmic alpha sinusoidal waves in the frontotemporal regions can provide indications of anti-NMDAR encephalitis [5–9].

Here, we report EEG and magnetoencephalography (MEG) data on EDB from a female patient with psychosis and reduced responsiveness. We address two questions: Does EDB in patients vary with open or closed eyes or with sleep-wake cycles? Do delta waves and beta activity originate in the same brain regions? The EEG data were used to elucidate the relationship between EDB and open or closed eyes and between EDB and occipital alpha rhythms. The MEG data were collected and evaluated using time-frequency analysis and magnetic source location to analyze the delta wave and beta activity.

## Case presentation

**Fig. 1:**
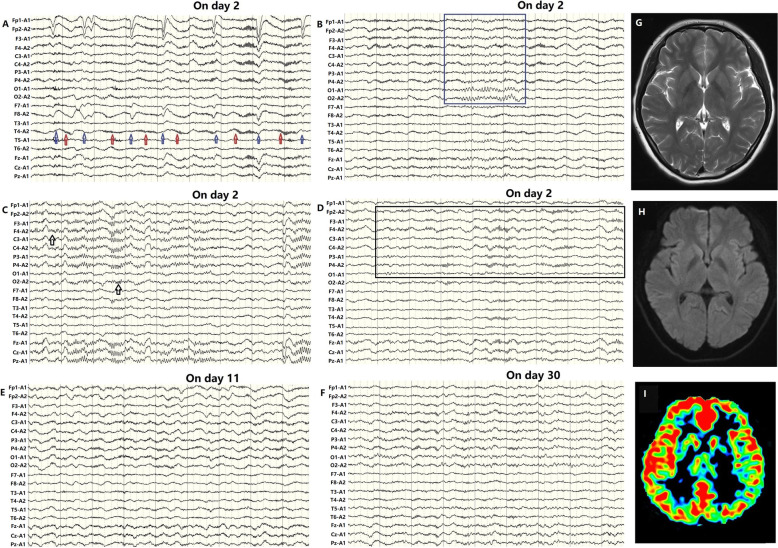
Extreme delta brush (EDB) on the first electroencephalography and magnetic resonance imaging. The beta activity of EDB weakened when the patient closed her eyes (**a**; blue arrow) and the occipital alpha rhythms occasionally reappeared (**b**; blue box). Conversely, the beta activity was enhanced when the patient opened her eyes (**a**; red arrow). EDB markedly weakened during stage II non-rapid eye movement (NREM) sleep (**c**) and was enhanced during slow-wave sleep (**d**). The background activity of the second EEG on day 11 was worse than that on day 2, and EDB was observed in bilateral brain regions (**e**). On day 30, the background activity in the third EEG recording was better than that on day 11. Compared to the initial EDB pattern on day 2, the EDB in the right brain regions was decreased (**f**). High-pass filter: 0.5 Hz; low-pass filter: 45 Hz. Sensitivity: 100µV/cm. FLAIR and T2 images were normal (**g, h**). Arterial spin labeling showed high blood flow in the right frontal and temporal regions (**i**)

The patient was a 16-year-old woman with no significant medical history. She presented at the Nanjing Brain Hospital with a fever of 37.5 °C, new-onset visual hallucinations and indifference. On day 2, the first video EEG recording showed EDB in the right brain regions, and normal occipital alpha rhythms were not observed (Fig. 1a). The patient was diagnosed with anti-NMDAR encephalitis according to EDB and received immunotherapy (glucocorticoid and immunoglobulin) immediately.

The beta activity within the EDB weakened when the patient closed her eyes (Fig. 1a; blue arrow), and the occipital alpha rhythms occasionally reappeared (Fig. 1b; blue box). Conversely, the beta activity was enhanced when the patient opened her eyes (Fig. 1a; red arrow). During sleep, EDB markedly weakened during stage II non-rapid eye movement (NREM) sleep (Fig. 1c) and was enhanced during slow-wave sleep periods (Fig. 1d).

On day 3 in the hospital, the patient experienced two seizures. Fluid-attenuated inversion-recovery (FLAIR)-weighted and T2 images were normal (Fig. 1g, h). Arterial spin labeling showed high blood flow in the right frontal and temporal regions (Fig. 1i). On day 4, indirect immunofluorescence technique (IIFT) results revealed NMDAR antibody titers of 1:100 in the CSF and 1:1000 in the serum. Gynecological sonography was normal.

The patient’s MEG data were recorded using a whole-head CTF 275-channel MEG system. MEG data were analyzed by time-frequency analysis, which was performed in each 5-s time window. Delta wave activity and beta activity were localized using magnetic source imaging. Detailed method involved in time-frequency analysis and magnetic source imaging has been described in our previous study [10–12].

**Fig. 2:**
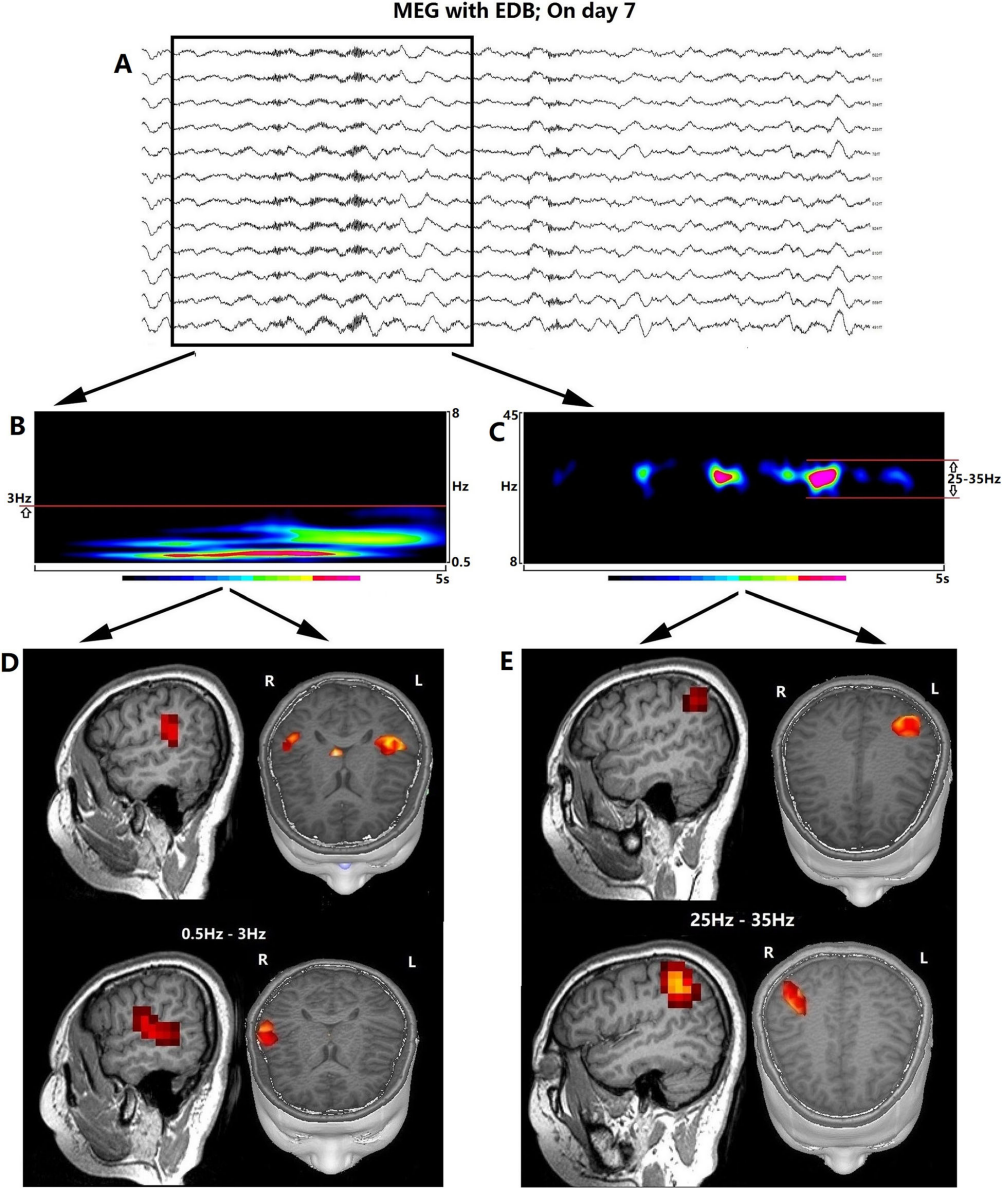
Extreme delta brush (EDB) on magnetoencephalography (MEG) and electroencephalography after treatment. MEG with bilateral EDB (**a**). Time-frequency analysis showing beta and delta activity (**b** and **c**). Magnetic source location showed that the delta activity originated in the bilateral superior temporal gyrus, the right middle temporal gyrus, the right inferior frontal gyrus, and the left inferior parietal lobe (**d**). Magnetic source location showed that the beta activity originated in the bilateral superior parietal lobe (**e**)

On day 7 the MEG results showed EDB evolving from the right brain regions to bilateral brain regions (Fig. 2a). Time-frequency analysis showed the beta activity varying from 25 to 35 Hz (Fig. 2c). The magnetic source location showed the beta activity originating from bilateral superior parietal lobes (Fig. 2e). However, the delta wave originated from the bilateral superior temporal gyri, the right middle temporal gyrus, the right inferior frontal gyrus, and the left inferior parietal lobe (Fig. 2d). On day 11, the background activity in the second EEG recording was worse than that on day 7 (Fig. 1e), and EDB was observed in bilateral brain regions (Fig. 1e).

After immunosuppression (immunoglobin, methylprednisolone and mycophenolate mofetil) and antiepileptic treatment (oxcarbazepine), the woman improved. On day 30, the background activity in the third EEG recording was better than that on day 11 (Fig. 1f). Compared to the first signs of EDB on day 2, the EDB indications in the right brain regions were obviously decreased (Fig. 1f). On day 38, the second serum antibody titer was 1:100. On day 40, the patient was discharged.

## Discussion and conclusion

EDB is regarded as a potential marker for anti-NMDAR encephalitis [4, 6–9], and contributed to the diagnosis of anti-NMDAR encephalitis in this patient. In previous studies, EDB has been observed primarily in women with CSF anti-NMDAR antibody titers at least 1:10 [7, 8]. The CSF antibody titer of this female patient was 1:100. Five days after disease onset, the first EEG recording showed EDB in the right brain regions (Fig. 1a-d). The increasing beta/delta power ratio might be an indication of anti-NMDAR encephalitis [5].

With deterioration due to the disease, EDB progressed from occurring only in right brain regions to occurring in bilateral brain regions (Fig. 2a and e). After immune and antiepileptic treatment, the woman improved. Signs of EDB in the right brain regions obviously decreased (Fig. 2f). EDB was observed only in the peak disease stage and continued to be a useful marker of disease activity and a tool for monitoring treatment response and relapses, through EDB resolution with clinical improvement [4, 7, 9].

We observed that the beta activity weakened when the patient closed her eyes (Fig. 1a; blue arrow). Why can the occipital rhythms inhibit beta activity in EDB (Fig. 1b; blue box)? The beta activity was located in the bilateral superior parietal lobe (Fig. 2d), which is adjacent to the occipital lobe. Thus, the occipital rhythms might propagate forward and weaken the beta activity in the superior parietal lobe. In a previous study, epileptic discharges that arose in the parietal cortex were observed to propagate forward widely via multiple fascicular pathways resulting in clinical features [13]. Therefore, the beta activity could be transmitted forward to the frontotemporal lobes (Fig. 2d) and superimposed with delta activity to form EDB on EEG.

EDB appears to be modulated by sleep. EDB weakened markedly during stage II NREM sleep and was enhanced during slow wave sleep. Unfortunately, rapid eye movement (REM) sleep was not observed during the EEG recordings. Dhruv et al. described a case of NMDAR encephalitis in which the patient entered REM sleep with an absence of EDB, which suggested that the antiepileptic effect of REM sleep could also affect EDB in NMDAR encephalitis [14].

In conclusion, EDB is useful as a marker of disease activity and as a tool for monitoring treatment response, and the pattern can be disturbed by eye opening and closing, by occipital alpha rhythms and by sleep-wake cycles.

## Data Availability

Data sharing is not applicable to this article as no datasets were generated or analyzed during the current study.

## Abbreviations

EEG: Electroencephalography
MEG: Magnetoencephalography
EDB: Extreme delta brush
Anti-NMDAR: Anti-N-methyl-d-aspartate receptor
